# AI-assisted, human-in-the-loop pipeline for converting historical documents into structured database: a feasibility study of environmental application in Michigan

**DOI:** 10.1080/2833373x.2026.2703318

**Published:** 2026-07-29

**Authors:** Qiong Zhang, Trueman Wu, Yousuf Hussain, Brendan F. O’Leary, Carol J. Miller, Mei Lu

**Affiliations:** a Department of Public Health Sciences, Henry Ford Hospital, Detroit, MI, USA; b Center for Leadership in Environmental Awareness and Research, Wayne State University, Detroit, MI, USA; c Henry Ford Health + Michigan State University Health Sciences, Detroit, MI, USA; d Civil and Environmental Engineering, Wayne State University, Detroit, MI, USA; e Department of Biological Sciences, Wayne State University, Detroit, MI, USA

**Keywords:** AI-assisted NLP pipeline, brownfield, environmental report digitization, FAIR

## Abstract

Most states have environmental guidelines or legislation that require any known site of subsurface contamination to undergo a baseline environmental assessment to document contaminant types, concentrations, and subsurface characterization (soil types, groundwater levels, etc.). The resulting publicly available documents are essential in understanding past contamination sources and the potential for future transport or exposure from these sites. Most states have an overwhelming archive of such documents that remain in only paper format, while newer documents are often available digitally. The older documents are very hard to evaluate and use in an efficient manner. Chemical data embedded in the documents were generated by multiple laboratories in varying formats, making them difficult to integrate and use for data analysis and reporting. There is an unmet need to systematically and efficiently extract the chemical data and ensure that the data are both human-readable and machine-readable and meet the principles of FAIR (Findable, Accessible, Interoperable, and Reusable). This study aimed to evaluate the feasibility of an AI-assisted, human-in-the-loop pipeline for extracting, reconstructing, harmonizing, and standardizing tabular chemical data from legacy environmental documents under real-world conditions. This study developed CLEAR-Extract, a scalable, flexible, and modular pipeline integrating optical character recognition, AI-assisted table reconstruction, and structured human quality assurance. Two pipeline configurations—a single-AI configuration emphasizing flexibility and a dual-AI configuration emphasizing efficiency and stability—were applied sequentially to historical brownfield documents from Michigan. Feasibility was assessed at the address level based on successful reconstruction of qualifying chemical tables, correct exclusion of non-qualifying documents, identification of true processing failures, and the degree of required human intervention. Across 99 candidate addresses, the pipeline successfully processed 98% of sites by either reconstructing qualifying chemical concentration tables or correctly excluding documents lacking relevant data. Only two addresses were classified as true failures due to severe document quality limitations. The resulting harmonized database captured volatile organic compound measurements spanning multiple decades and heterogeneous reporting formats, standardized into a common schema suitable for downstream environmental health analysis. This paper demonstrated that CLEAR-Extract, an AI-assisted, human-in-the-loop pipeline, could achieve high reconstruction success from heterogeneous legacy environmental documents. The approach also demonstrated a scalable and transferable framework for converting unstructured historical documents into a FAIR-aligned, analyzable database for environmental health research and regulatory decision-making.

## 1. Introduction

The advent of computers has led to a transformative era for information preservation. This digital revolution has significantly altered archival methods, leading to the widespread digitization of documents. All entities, such as institutions, organizations, and governments, have implemented strategies to digitize vast collections, enhancing public access and preserving historical records (National Archives 2025). Environmental documents, particularly those documenting chemical hazards associated with human health risks, represent a critical component because they are used to inform a broad range of risk-related decisions across the agency and a critical component of this broader digitization effort. Many of these documents are centered at brownfield sites. According to the U.S. Environmental Protection Agency (2025), brownfields are properties where expansion, redevelopment, or reuse may be complicated by the presence or potential presence of hazardous substances, pollutants, or contaminants.

Despite the large volume of environmental assessment documents available, much of the information remains embedded in unstructured and heterogeneous formats, limiting its usability for large-scale analysis and evidence-based decision-making. Berman et al. (2022) provided a comprehensive examination of brownfield sites redevelopment in the United States and noted that less than 7% of the estimated 450,000 brownfield sites nationwide had been fully assessed or remediated despite federal and state initiatives. This gap represented an emergent public health and environmental health crisis. Among the limitations highlighted in their analysis, the largest drawback was the data-limited decision-making within the regulatory framework, particularly the U.S. Environmental Protection Agency (EPA)’s Brownfields Program. It highlighted a critical gap in data availability and accessibility, as many documents were scanned and stored as image files only. The information they contained remained static and inaccessible to modern computation workflows. The lack of an integrated, accessible, harmonized, and analyzable environmental database continues to constrain the ability of regulators, researchers, and communities to make data-driven, informed decisions. Recent state-level initiatives have begun addressing this gap. For example, in Michigan, the Remediation Information Data Exchange (RIDE) system was launched by the Department of Environment, Great Lakes, and Energy (EGLE) in 2019 to modernize the submission and management of environmental remediation data statewide (EGLE, 2019). Electronic submittals were first enabled in 2022 and later expanded in 2024 to all facilities, representing a recent program-wide rollout of digital reporting intended to standardize, centralize, and enhance the accessibility of remediation records across the state (EGLE, 2022, 2024).

Artificial intelligence (AI) has increasingly been applied to environmental monitoring, particularly in areas of air and water quality, climate change, soil mapping, and disaster management, to underscore the transformative potential of new technologies for sustainable environmental management (Alotaibi and Nassif 2024). Recognizing these opportunities and challenges across environmental domains, the EPA hosted a workshop in May 2022 to explore how advances in AI and data science could enhance human health risk assessment and environmental data utilization (NASEM 2022). Recent advances in natural language processing (NLP), computer vision, and intelligent document processing have further enabled the possibility of having text extraction, prompt engineering, and data quality evaluation with Generative AI (Watford, Schoeff, and Ford 2025). More Machine Learning (ML) tools have been developed to extract data and synthesize languages in systematic chemical assessments (Angrish et al. 2024).

However, existing approaches often struggle to handle the heterogeneity, inconsistency, and scale of legacy environmental documents, particularly when extracting structured tabular chemical data across diverse formats. Experts in the EPA workshop (2022) emphasized that while AI tools can assist in identifying and filtering components within environmental documents, significant challenges persist in extracting, standardizing, and integrating chemical data. Additionally, these chemical data are often embedded within heterogeneous formats originating from multiple laboratories, making systematic extraction and downstream analysis difficult. As a result, there is currently no established protocol for transforming legacy environmental documents into structured, analyzable datasets. The extent to which AI can be used to enhance chemical data extraction remains largely unknown.

Volatile organic compounds (VOCs), a common contaminant at legacy brownfield sites (Miller et al. 2020), are important determinants of maternal and offspring health, with established links to preterm birth and adverse health outcomes (Cassidy-Bushrow et al. 2020). VOCs have contaminated shallow soils and groundwater of post-industrial cities at residential, commercial, and industrial properties, leading to potential exposures via vapor intrusion (Miller et al. 2020; Squillace, Moran, and Price 2004). The Center for Leadership in Environmental Awareness and Research (CLEAR) is a National Institute of Environmental Health Sciences (NIEHS) P42 Superfund Center at Wayne State University dedicated to understanding and mitigating adverse health problems that have been associated with environmental pollution (https://clear.wayne.edu/). CLEAR unites experts across multiple disciplines including public policy, environmental health sciences, environmental engineering, and public health to address the complex challenges posed by brownfields redevelopment.

The digitization of historical environmental records also highlights an urgent need for robust data stewardship frameworks in environmental health domain that ensure long-term usability and transparency. Wilkinson et al. (2016) developed the FAIR guiding principles—Findable, Accessible, Interoperable, and Reusable—to regulate the standardized, machine-readable data structure that support cross-study synthesis and computational integration. In environmental science, FAIR approaches have been increasingly recognized as a gold standard for managing complex, heterogeneous datasets, such as community-centric metadata, geospatial records, and regulatory assessments (Cannon, Kelly, and Freeman 2022; Crystal-Ornelas et al. 2022; Kinkade and Shepherd 2022).

The objective of this feasibility study was to develop and evaluate a scalable, AI-assisted, human-in-the-loop pipeline for extracting, reconstructing, and harmonizing VOC data from heterogeneous legacy environmental documents into a structured, analyzable database under real-world conditions. Specifically, we aimed to: (1) design a modular, scalable document-to-database pipeline that integrates optical character recognition (OCR), natural language processing (NLP), and structured human quality assurance; (2) assess the feasibility of this pipeline across highly heterogeneous historical documents by quantifying successful processing, failure modes, and required human intervention; and (3) demonstrate how the resulting structured data can be standardized to align with FAIR principles. This study emphasizes feasibility and pipeline structure rather than formal benchmarking of AI model performance.

## 2. Material

This study collected environmental remediation documents from EGLE’s RIDE database. RIDE serves as the state’s centralized digital repository for environmental remediation documents submitted under Michigan’s cleanup and redevelopment laws—primarily Part 201 (Environmental Remediation) and Part 213 (Leaking Underground Storage Tanks) of the Natural Resources and Environmental Protection Act (NREPA), 1994 PA 451. It houses documents such as Baseline Environmental Assessments, No Further Action Reports, and Response Activity Plans under Part 201, as well as Initial and Final Assessment Reports, Corrective Action Plans, and Closure Reports under Part 213. These submissions, often accompanied by maps, boring logs, laboratory data, and EGLE correspondence, are uploaded electronically through the MiLogin for Business portal and stored in standardized digital formats to facilitate regulatory review, data integration, and public transparency. While these materials are electronically accessible, their quality and structure remain highly variable.

These legacy documents—often stored as unstructured PDFs or scanned documents—contained valuable site-specific information on contaminant concentrations, sampling locations, media types (soil, groundwater, air), and laboratory analytical results. In this feasibility study, we sampled 99 addresses with more than 1000 environmental documents from 7,539 addresses in the region of MI. All documents were related to chemical concentration in soil, water, and air samples. The documents spanned from the 1980s to the 2020s and were issued by multiple laboratories and regulatory sources, including the Michigan Department of Environment, Great Lakes, and Energy (EGLE), the Department of Environmental Quality (DEQ), AKT Peerless, and FiberTech Environmental Services.

Due to the absence of regulation on document format, considerable heterogeneity existed across these documents. File formats varied from digitally generated Word documents to scanned images or photocopies of paper records. The visual appearance of the documents also differed markedly, with some pages printed on white, blue, or red due to variations in templates or scanning conditions. The content itself ranged from narrative paragraphs and regulatory forms to detailed laboratory data sheets/tables, often coexisting within a single page. Even among tabular data, wide structural variability was observed with some tables constructed in neatly formatted, while others were hand-filled or annotated, containing inconsistent column alignments and unit representations. This high degree of heterogeneity across documents underscored the necessity of an AI-assisted framework that was capable of automatically detecting, parsing, and reconstructing environmental data from these legacy sources.

## 3. Method

### 3.1. Study design

This study was designed as a feasibility evaluation of an AI-assisted, human-in-the-loop pipeline for extracting and harmonizing VOC data from heterogeneous legacy brownfield chemical assessment documents. The unit of analysis was the address, defined as a unique site location associated with one or more environmental documents. The feasibility was assessed based on the pipeline’s ability to correctly identify relevant reports, reconstruct chemical tables when present, and harmonize extracted data into a predefined common schema suitable for downstream environmental health research. The target output of the pipeline was a standardized VOC-related database comprising 10 core variables including File FMT (file formats), File ID (unique identifier for the file), page number (page number of the file), Parameter (chemical name $), unit, result, range of the lower detection (RL), Dilution, date of sample prepared (Date PREP) and data of sample processed/analyzed (Date Analyzed) (Table 1). The schema was designed to accommodate heterogeneous reporting formats while preserving essential analytical context required for downstream environmental health analyses.

### 3.2. Pipeline overview and operational definitions

**Figure 1.**
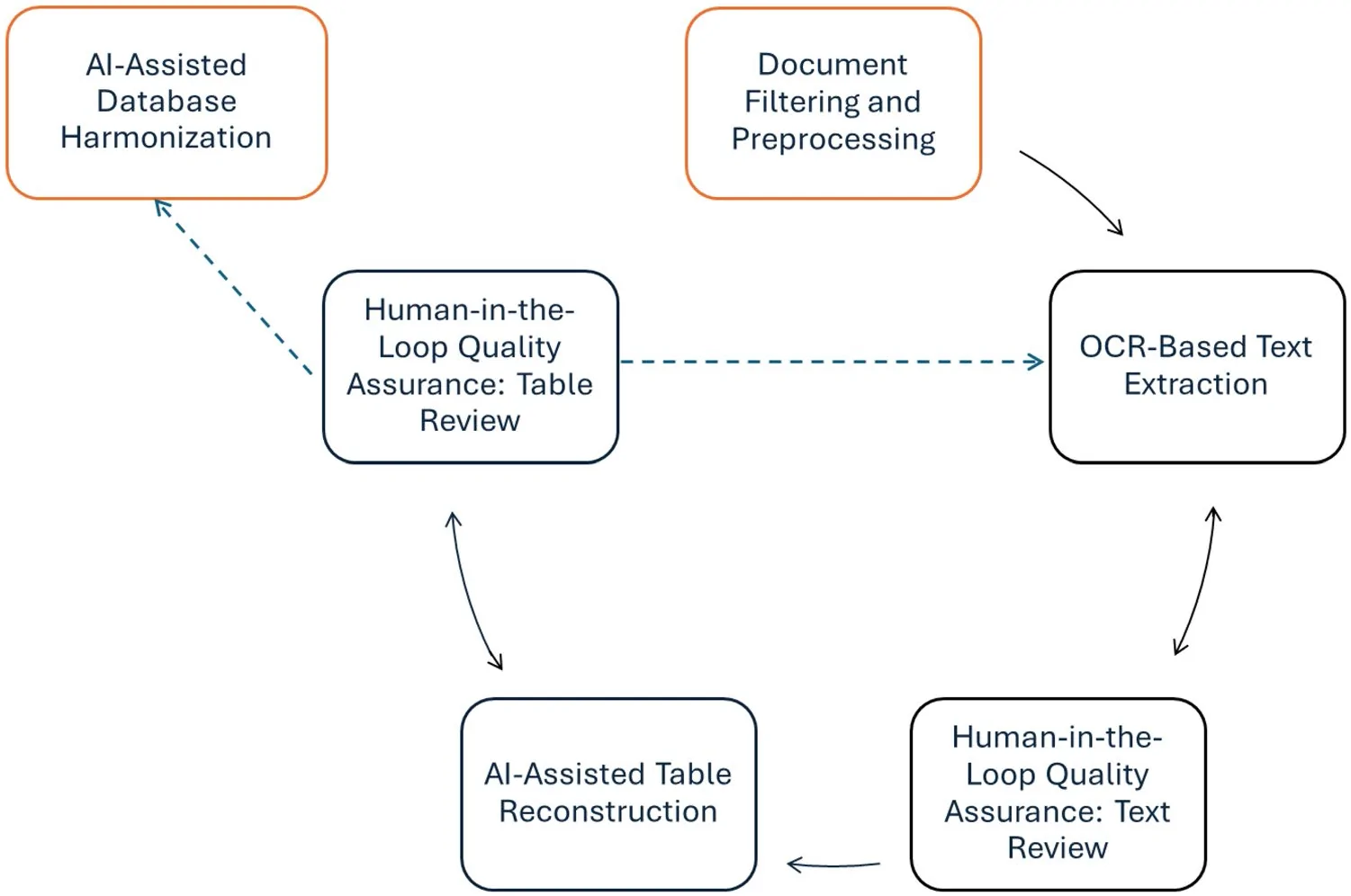
Conceptual overview of the CLEAR-Extract AI-pipeline.

CLEAR-Extract is a modular, AI-assisted pipeline integrating OCR, NLP, and structured human quality assurance (QA) at multiple checkpoints to convert unstructured environmental reports into standardized tabular database (Figure 1). The design followed an iterative, semi-circular design that supports flexibility while minimizing errors, including document filtering, document page level downsizing, text extraction, table reconstruction, quality assurance checkpoints, and final data harmonization into a common schema.

Human-in-the-loop oversight was embedded at predefined checkpoints to evaluate document relevance, assess OCR adequacy, validate reconstructed tables, and determine whether reprocessing was required. When outputs did not meet predefined quality criteria, the workflow supported iterative reprocessing. Pipeline execution terminated when either a valid chemical table was successfully reconstructed and harmonized, or when all associated documents were correctly determined to lack relevant chemical tables.

The workflow begins with document filtering and preprocessing of candidate environmental documents, followed by OCR-based text extraction, AI-assisted table reconstruction, and harmonization into a standardized database. Human-in-the-loop quality assurance is embedded at both the text and table stages to support iterative review and reprocessing when needed (double-headed arrows). Table detection is applied selectively as a part of processing in Experiment 2 only, as described in Section 3.3.

The terminology and operational definitions were standardized throughout this study. The term “pipe-line” refers to the complete, scalable, modular document-to-database system integrating OCR, NLP-based table reconstruction, and structured human quality assurance. “AI-assisted” indicates that AI tools were used to perform specific tasks under predefined rules, with human oversight governing decision-making, error resolution, and validation at each stage (human-in-the-loop). An address was defined as a unique site location associated with one or more environmental documents. A legitimate address was defined as an address for which at least one associated report contained potentially relevant chemical testing information. An address was considered successfully processed if either (1) at least one report associated with that address yielded a reconstructed chemical table that met predefined structure and variable criteria and could be harmonized into the common data schema, or (2) the pipeline correctly determined that none of the associated documents contained relevant chemical tables meeting inclusion criteria. Address was considered unsuccessfully processed only when relevant chemical tables were present but could not be reliably identified or reconstructed.

Prompt engineering followed structured templates designed to preserve tabular relationships and enforce schema consistency during reconstruction. Table detection and reconstruction steps were guided by empirically defined criteria that were established through iterative testing across representative document samples. Rule-based validation was applied to ensure alignment between extracted column headers and corresponding data entries. All source documents were publicly available environmental documents and contained no identifiable or sensitive information. AI services were used solely for document processing.

### 3.3. Experiment configuration

This study deployed two pipeline configurations that shared the same underlying end-to-end design (Figure 1) but differed in the AI platforms and preprocessing strategies. These configurations were applied sequentially to the same document corpus and were not intended as independent experiments. They were alternative instantiations of the same design concept to examine tradeoffs between flexibility, efficiency, and human intervention.

#### 3.3.1. Experiment 1: Single-AI pipeline configuration

**Figure 2.**
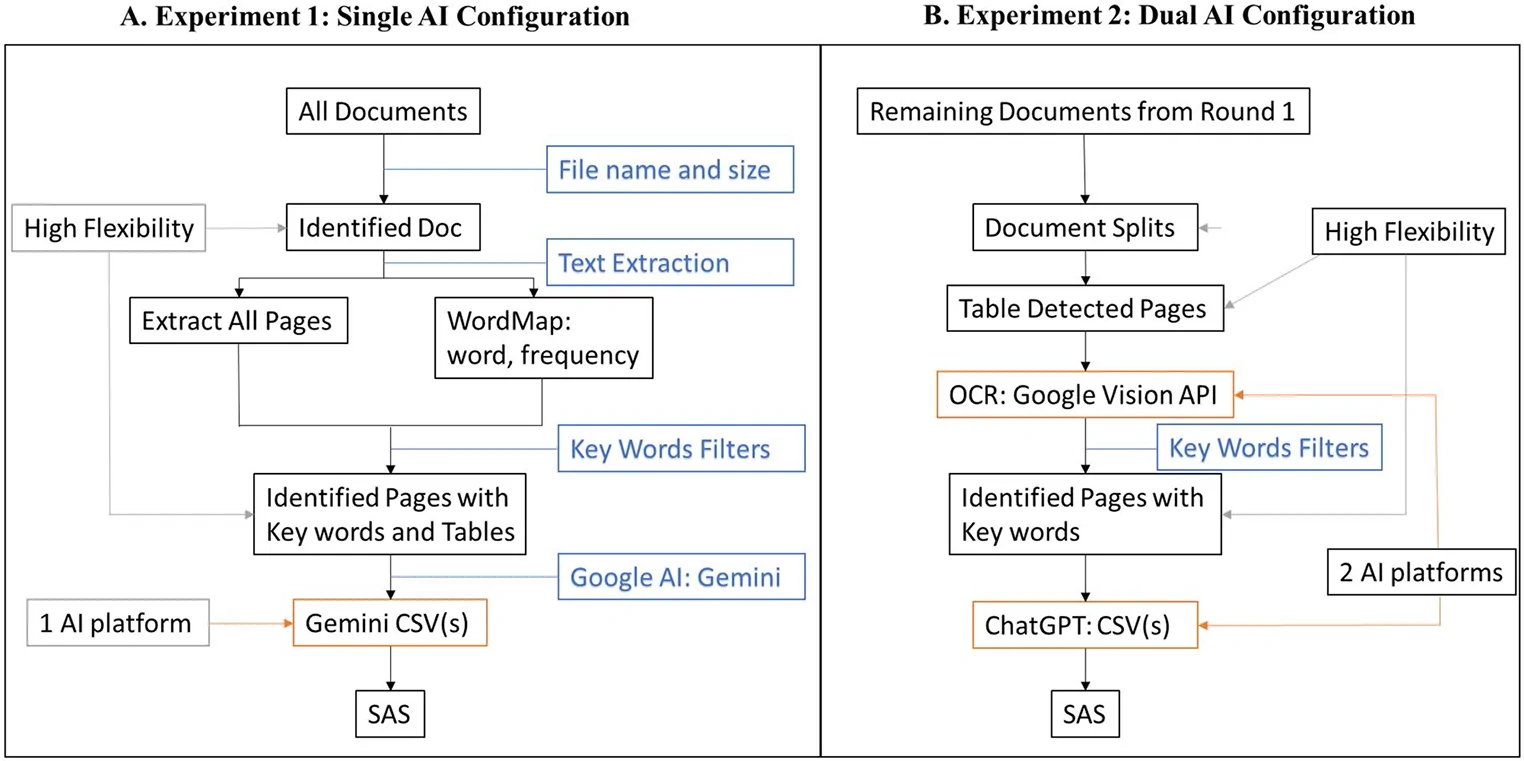
Two AI-assisted pipelines configuration of the CLEAR-Extract.

Experiment 1 implemented a single-AI configuration for the CLEAR-Extract pipeline, prioritizing flexibility, rapid iteration, and configurability (Figure 2A Experiment 1: Single AI Configuration). In this configuration, OCR was performed using an open-source Python Tesseract OCR package (Version 5) (Smith 2007), while a single AI platform Google Gemini Pro (Version 1.0) (Google, 2024) was used for downstream text interpretation and table reconstruction. The use of Tesseract OCR tool in Experiment 1 allowed for higher control of OCR processing parameters and facilitated interactive adjustment based on observed outputs.

Pipeline execution in Experiment 1 consisted of document identification based on file name patterns and file size, page-level splitting, OCR-based text extraction using Tesseract, keyword-based filtering to identify candidate pages containing VOC-related information, and Gemini-assisted reconstruction of chemical tables into structured CSV outputs. Keywords filters were chemical variables that were selected based on their relevance to environmental health investigations to the CLEAR researchers, including benzene, toluene, ethylbenzene, xylene, trichloroethylene, tetrachloroethylene, m-xylene, o-xylene, p-xylene, xylenes, total xylenes, trichloroethene.

Human-in-the-loop was applied at beginning document reviewing and preparation stages as well as multiple assurance points, including assessment of OCR output adequacy and evaluation of reconstructed table structure and quality. When reconstructed outputs could not meet predefined quality criteria, such as missing required variables, incomplete chemical pollutants, or unreadable character, reprocessing was initiated using alternative OCR configurations or revised reconstruction prompts. Experiment 1 was conducted on a subset of addresses (n = 99) from the master document corpus as an initial feasibility assessment of the pipeline under a single AI configuration.

#### 3.3.2. Experiment 2: Dual-AI pipeline configuration

Experiment 2 implemented a dual AI configuration for the CLEAR-Extract pipeline, emphasized processing stability and success for documents that were challenging to handle under the single-AI configuration (Figure 2B Experiment 2: Dual AI Configuration). Experiment 2 did not constitute an independent experiment conducted on a separate dataset. It represented a refined configuration of the same pipeline applied sequentially to addresses that were not successfully processed in Experiment 1.

In this configuration, page-level table detection was first applied to identify candidate pages containing tabular context. The LayoutParser (Shen et al. 2021) model was applied to each page to perform table detection and only pages with tables were retained for the following OCR step. The OCR was then applied using commercial Google Vision API OCR service (Version 3.13) (Google, 2024) to extract text from selected pages. The AI-assisted table reconstruction was subsequently conducted using large language model ChatGPT (Version 4.1) (OpenAI 2024) to transform extracted text into structured tabular format.

Human-in-the-loop was retained at the same stages as in Experiment 1, including evaluation of table detection, assessment of OCR output, and validation of reconstructed table structure. Reprocessing decisions were made when outputs could not meet predefined quality criteria as in Experiment 1. By separating OCR and table reconstruction to different specialized AI platforms, Experiment 2 assessed the feasibility of a dual-AI configuration for table reconstruction while addressing document heterogeneity.

This figure illustrates two sequentially implemented configurations of the CLEAR-Extract workflow. Both configurations follow the same canonical end-to-end workflow shown in Figure 1 but differ in AI platforms and preprocessing strategies. Experiment 1 employs a single AI configuration emphasizing flexibility through open-source OCR Python package (Tesseract) and Gemini, while Experiment 2 adopted dual AI configuration combining page-level table detection, Google Vision-based OCR, and ChatGPT-assisted table reconstruction. Human quality assurance and feedback loops are embedded in both configurations to guide reprocessing decisions and ensure downstream suitability,

### 3.4. Evaluation criteria and feasibility outcomes

#### 3.4.1. Human evaluation of table detection (Experiment 2 only)

Table detection was introduced in experiment 2 to reduce processing burden by restricting OCR to pages containing tables only. Reviewers first identified 3–5 representative table formats that appeared frequently across documents and manually verified whether the table-detection algorithm identified these tables when applied to multiple documents. Additional spot checks were also conducted by randomly sampling processed PDFs and comparing detected table pages against the original documents to confirm that tabular content was systematically caught.

#### 3.4.2. Human evaluation of OCR outputs

Human-in-the-loop evaluation of OCR outputs was conducted through structured quality assurance procedures. Four researchers independently performed evaluations by reviewing randomly selected subsets of addresses (*n* = 10) processed by the pipeline. For each selected address, evaluators examined the OCR output alongside the original source documents, reviewing page by page to determine whether OCR outputs were suitable for downstream table reconstruction.

OCR outputs were evaluated based on multiple operational criteria, including page coverage, text legibility, and character readability. Evaluators assessed whether key content, such as chemical names, numerical values, units, and tabular context, was readable and understandable. OCR outputs containing excessive unreadable characters, severe text missing, or systematic character misrecognition that heavily compromised interpretability was classified as inadequate.

For Experiment 1, which did not incorporate page-level table detection, all pages associated with selected documents underwent OCR processing and were expected to yield texts. Pages that were not OCR-processed or no text-yielded under this configuration were classified as processing failures attributable to OCR execution. For Experiment 2, which incorporated table detection prior to OCR, evaluation involved distinguishing between pages correctly excluded due to the absence of tabular content and pages that contained tables but were not OCR-processed due to OCR execution failure. This distinction enabled different decisions between correct pipeline behavior and re-processing consideration.

#### 3.4.3. Human evaluation of table reconstruction outputs

Reconstructed tables generated by the pipeline were evaluated through independent human review by the same four researchers. Evaluators compared reconstructed CSV outputs against the original source documents to assess structure integrity, contextual validity, and content readability. Evaluation criteria focused on whether reconstructed tables preserved the original table structure, aligned column headers with corresponding data entries, and conformed to expected data types defined in the common schema (e.g., chemical names are character, unit are character, concentration or density or result are numeric, sample date as date, etc.). Quantitative performance metrics such as precision and recall were not computed due to the absence of a fully annotated gold-standard dataset for benchmarking.

CSV files were first evaluated for whether they preserved a tabular structure. A file was classified as “good” if the header row could align well with all data rows. In some cases, reconstructed table contained an extraneous empty padding row or column. If removing is restored consistency, the file was classified as “good with modification”. Outputs that did not initially exhibit a consistent tabular structure were further assessed for whether rule-based cleaning could restore tabular validity. Cleaning attempts included removal of non-analytical rows or columns (e.g., column containing “%”), ignoring a small number of trailing summary rows (e.g., the last three rows that contain no chemical information), or separating documents containing multiple disconnected tables based on empty padding rows and retaining the final section when it contained the target chemical table. Outputs that could achieve consistent table structure after these cleaning procedures were classified as “bad but needs clean”. Documents that remained non-tabular after these cleaning procedures were classified as “bad and unclean” and excluded from downstream harmonization.

Content-level evaluation emphasized readability and interpretability. Evaluators assessed whether (1) column headers aligned with corresponding entries and conformed to expected data types defined in common schema; and (2) reconstructed values were free of systematic character misrecognition (e.g., confusion between “L” and “I” or between “4” and “1”), missing entries, or contextually implausible values. Particular attention was given to chemical name and concentration-related columns, which were critical for downstream environmental health analyses. Columns deemed ancillary to analytical objectives, such as laboratory personnel identifiers or analytical method descriptors, were evaluated with lower stringency and were excluded from the final harmonized database when not consistently presented in all documents.

Reconstructed tables meeting predefined structural and contextual criteria were classified as suitable for harmonization. Tables exhibiting misaligned columns, missing required variables, unreadable content, or substantial errors were either reprocessed using alternative reconstruction prompts or flagged as failure for further human decision.

## 4. Results

### 4.1. Address-level corpus overview

The master brownfields database comprised 7539 unique addresses. The research team selected 99 sites based on three criteria: (1) inclusion in EGLE’s Part 201 or Part 213 programs, (2) availability of relevant documentation associated with the sites, and (3) location within an area of interest of environmental health studies for the CLEAR research team—specifically, Wayne County, Michigan. All 99 addresses entered the single-AI configuration pipeline (Experiment 1) and were subsequently evaluated under a refined dual-AI configuration (Experiment 2), as needed.

### 4.2. Feasibility assessment: Experiment 1-single AI configuration

**Figure 3.**
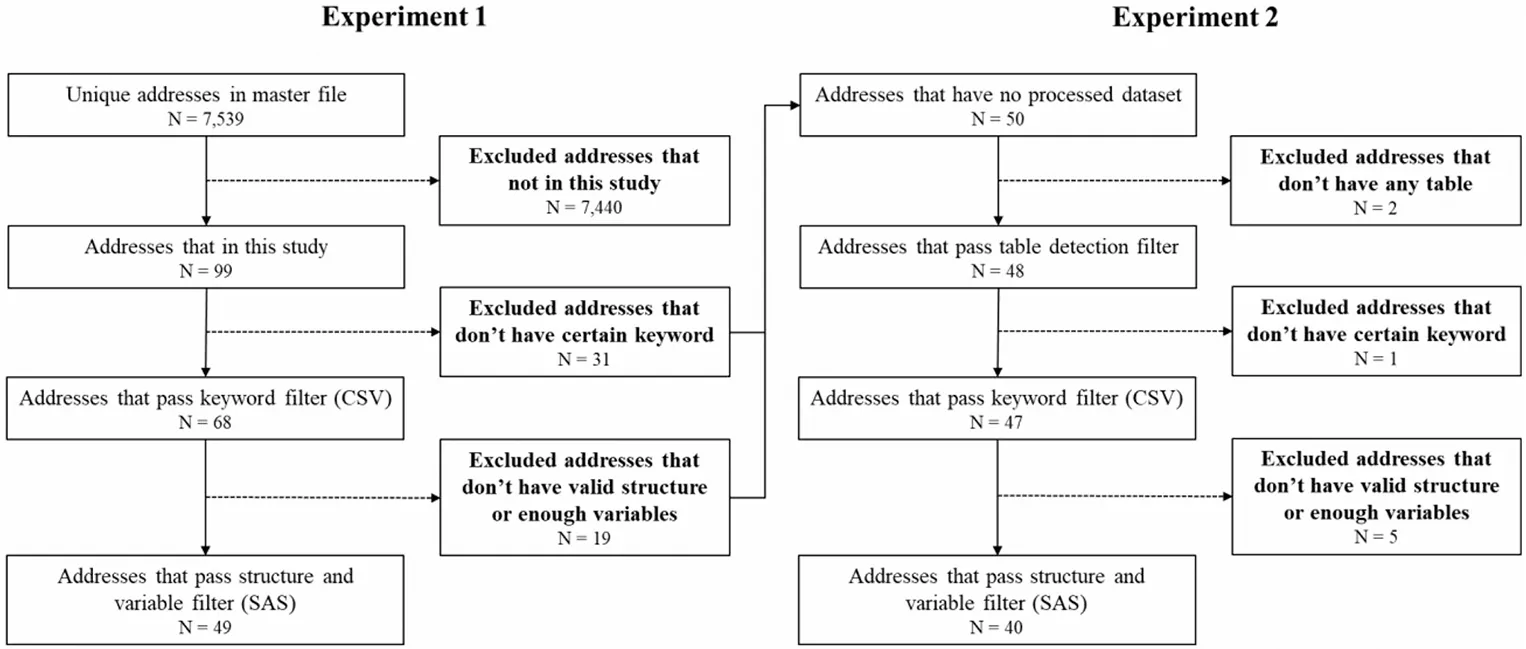
Processing outcomes across Experiment 1 and Experiment 2.

Under the single-AI configuration, all 99 selected addresses were processed. Following OCR, keyword filtering, table reconstruction, and structured human evaluation, 49 addresses yielded reconstructed chemical tables meeting predefined structural and variable criteria and entered the data harmonization stage (Figure 3). The remaining 50 addresses were carried forward for further evaluation using the dual-AI configuration.

Figure 3 illustrates address-level processing outcomes across the two pipeline configurations, distinguishing successful table reconstruction, correct exclusion of non-qualifying documents, and true processing failure.

### 4.3. Feasibility outcome: Experiment 2-dual AI configuration

Experiment 2 was applied to the 50 addresses that did not enter data harmonization under Experiment 1 (Figure 3). Under the dual-AI configuration, 40 addresses yielded reconstructed chemical tables meeting predefined harmonization criteria and entered the harmonization stage.

Among the remaining 10 addresses, 8 were correctly excluded by the pipeline, including addresses for which no tabular content was identified during the table-detection stage (*n* = 2), addresses containing tables without relevant chemical measurements following keyword-based filtering (*n* = 1), and addresses containing tabular chemical data but did not represent concentration documents and lacked required variables (e.g., unit, result, reporting limit, dilution, lab date), despite containing relevant chemical names (*n* = 3).

Only 2 addresses were classified as unsuccessfully processed. These addresses contained chemical concentration tables but could not be reliably reconstructed due to severe document quality limitations, including low-resolution scans and image degradation.

### 4.4. Failure modes and human evaluation

Unsuccessful processing outcomes were attributable to a limited set of document-level constraints identified through structured human-in-the-loop evaluation. Across both pipeline configurations, four researchers independently reviewed OCR outputs and reconstructed tables by comparing pipeline outputs against original source documents on a page-by-page basis, following predefined operational criteria (Section 3.5). Failure modes primarily included low-resolution scans and image degradation that prevented reliable OCR and table reconstruction.

In contrast, several addresses were intentionally excluded from data harmonization by pipeline, validating by human evaluation. These correct exclusions included documents without tabular content, having table but lacking relevant chemical names, and having both table and chemical names but not meet predefined structural or variable requirements for concentration reporting. Correct exclusion of these documents was also considered a success, as it prevented the generation of spurious structured data. Human-in-the-loop evaluation played a critical role in distinguishing between pipeline behavior and true processing failures.

### 4.5. Characteristics of the harmonized VOC database

**Figure 4.**
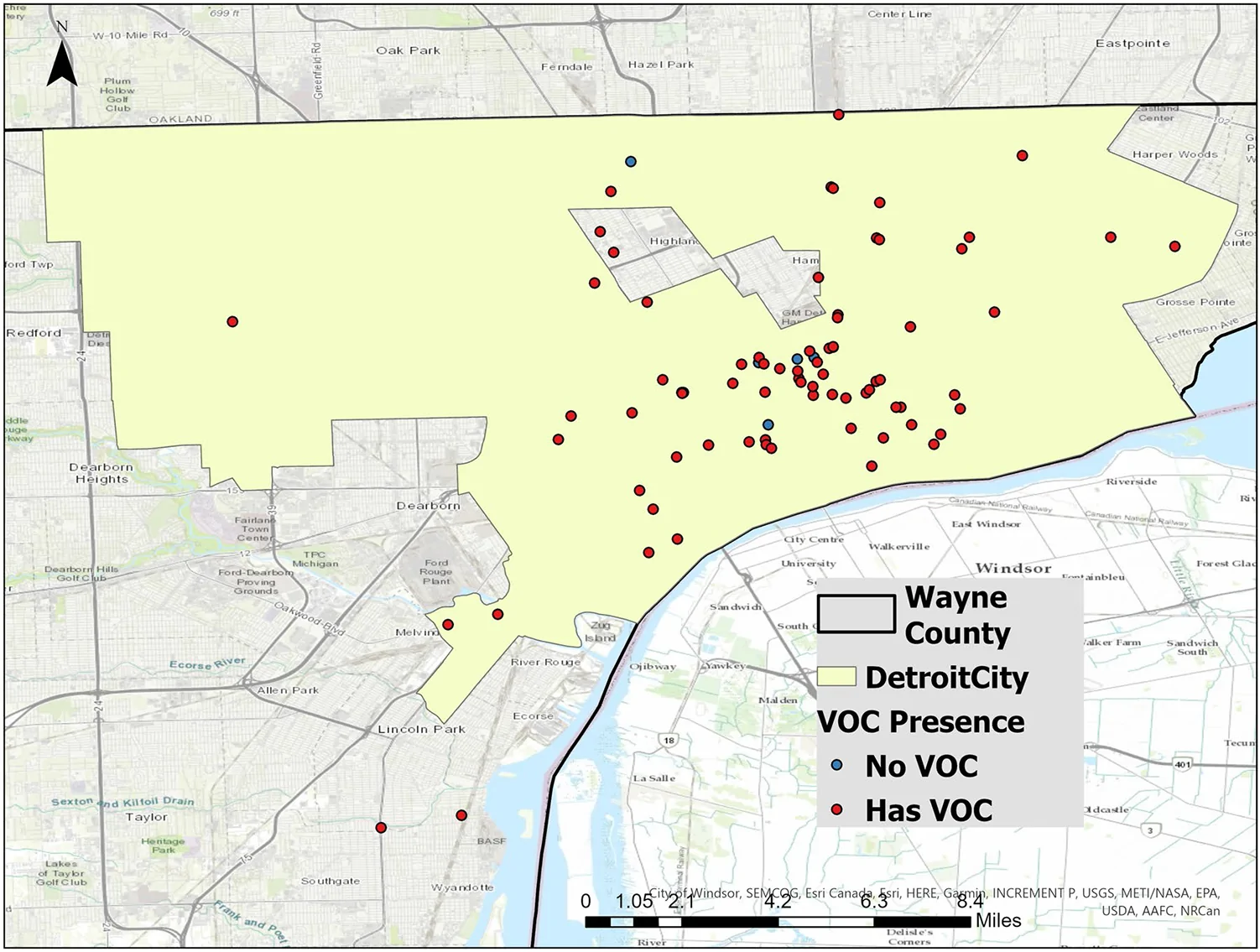
The spatial distribution of harmonized brownfield sites. *Red points represent VOC-evaluated sites, where volatile organic compound data tables were identified and extracted from available documents. Blue points denote non–VOC-evaluated sites, where either no VOC data were reported or the sites had not been assessed for VOC contamination.

Among addresses that entered data harmonization under either pipeline configuration, a total of 89 addresses containing 159 PDFs comprising 4270 pages contributed to the final data chemical database. Figure 4 illustrated the spatial distribution of these 89 addresses. Most sampling locations (red dots) represented sites where volatile organic compound (VOC) data were successfully extracted and harmonized from available documents, whereas blue dots indicated sites for which no extractable VOC information was identified. The spatial distribution reflected heterogeneity in data availability across sites within the study area.

Supplementary Table 1 summarizes address-level characteristics of the harmonized chemical data. Across 89 addresses, eight distinct chemical table formats were identified, reflecting heterogeneity in legacy reporting structures. Each address was assigned a table format category based on the predominant format observed in its associated documents. For each address, the total number of pages containing relevant chemical tables was recorded, along with total number of reconstructed rows aggregated across all qualifying tables. These reconstructed rows present individual chemical measurements extracted from multiple tables and pages associated with a given address. At the address level, the number of chemical-related PDF documents ranged from 1 to 8, corresponding to 1–416 pages containing relevant chemical tables and 10–16,937 reconstructed rows per address (Supplementary Table 1). The temporal coverage of reconstructed chemical data varied across sites, with dates ranging from 1982 to 2023.

The initial extraction identified 1099 unique chemical names, reflecting a mixture of full names, partial names, and misspelled or incorrect names. After two iterations of data cleaning, the number of unique chemical names was reduced to 373 including VOC chemicals (benzene, toluene, ethylbenzene, xylene, trichloroethylene, tetrachloroethylene, m-xylene, o-xylene, p-xylene, and trichloroethene).

Targeted VOCs measurements were observed in 84 of the 89 harmonized addresses (94%), accounting for 6.8% of all chemical records. Measurements below the limit of detection (LOD) were imputed as LOD/2, a commonly used approach in environmental epidemiology when the proportion of non-detects is low. The VOC abnormal rate was about 20% (Table 2). Summary statistics for observed VOC values (raw measurements) and derived variables (imputed values and detection indicators) were presented in Table 2. Differences between mean and median values across multiple VOC compounds indicated the presence of outliers and skewed distributions, which is very common for chemical measurements.

## 5. Discussion

### 5.1. Overview of key findings

This study demonstrated that a practical and scalable AI-assisted pipeline can convert legacy environmental documents into a structured, analyzable database tailored to specific research needs. By combining OCR, table reconstruction, and structured human oversight, the CLEAR-Extract pipeline successfully processed the vast majority of candidate addresses while correctly excluding non-informative documents. Importantly, feasibility was achieved through strategic integration of human effort at critical decision points. Together, these findings illustrate a scalable, feasible approach to transforming static environmental records into reusable data assets.

This study exclusively utilized publicly available environmental assessment documents obtained from an environmental organization (EGLE). The documents contained no personally identifiable information, protected health information, or other legally sensitive data. All data processed through commercial AI platform were limited to non-confidential, site-level environmental documents intended for public information.

AI platforms were used solely to support OCR or table reconstruction. No model training or fine-tuning was performed, and all outputs were subject to structured human review prior to being included in the harmonized database. The use of third-party AI tools complied with institutional data governance, ensuring that data handling was not subject to privacy, security, or regulatory risks.

### 5.2. Practical challenges and design tradeoffs

The development of the CLEAR-Extract pipeline required addressing multiple practical challenges inherent to legacy environmental assessment documents. An outstanding challenge was the extreme heterogeneity of historical documentation. Individual addresses were often associated with multiple documents produced by different laboratories over extended time periods. Each employed distinct reporting conventions, terminologies, and table structures. In many cases, only a subset of documents contained chemical concentration data relevant to the research objectives, while others included administrative summaries, qualitative descriptions, or non-chemical tables. This heterogeneity precluded rigid assumptions about document structure and necessitated a flexible, adaptive pipeline design. To address this challenge, human effort was employed. Four researchers independently viewed a random sample of 10–20 addresses, assessed the presence and the structure of chemical tables, verified the existence of required chemical information, and determined overall document relevance. Additionally, the key characteristics for each document, including file size, document name, and tester were recorded and summarized. Findings from this manual review informed the development of preliminary screening criteria for document selection, including minimal file size threshold (>1 kilobyte), minimal document length (>1 page), and the presence of specific patterns in filenames (e.g., “BEA”, “RI”).

The second major challenge involved balancing processing efficiency against data reliability. Environmental documents frequently exceeded hundreds of pages, yet only a small fraction contained relevant chemical tables. The open-source Python OCR package used in Experiment 1 showed that processing entire documents through OCR not only increased computational cost but also elevated the risk of context drift and error propagation. To address this challenge, in Experiment 2, the pipeline incorporated page-level table detection to selectively target pages most likely to contain relevant tabular data. This design choice reduced unnecessary processing while preserving the ability to recover meaningful information from diverse document layouts. These strategies decreased the risk of having the context drift and error propagation from processing long documents.

Quality assurance posed another challenge, particularly in distinguishing relevant chemical concentration tables from non-informative or misleading tabular content. Legacy documents often contained tables listing chemical names without quantitative measurements or summary tables lacking targeted information. Treating all detected tables as equally informative would undermine database integrity and inflated downstream errors. To address this challenge, the pipeline emphasized correct exclusion as an explicit design goal, allowing non-qualifying tables to be intentionally removed from further processing. This exclusion should not be considered a failure, but rather a necessary safeguard against generating spurious structured tables.

Finally, data harmonization required navigating substantial variation in all table structures and variable representations. Reconstructed tables varied widely in the number and naming of fields, units of measurement, and conventions for representing non-detects. In some documents, reconstructed tables contained dozens of variables requiring extensive transformation, while others might have fewer fields and required less cleaning. To address this challenge, the pipeline deferred strict standardization to later harmonization steps, where domain expertise could guide variable selection, unit conversion, and data cleaning. This tradeoff enabled the pipeline to remain adaptable to heterogeneous inputs while ultimately producing a consistent, analyzable dataset aligned with downstream environmental health research needs.

### 5.3. Human-AI collaboration and governance

This work aligned with broader scientific calls to transform static information into machine-readable and analyzable data resources. Bryan et al. (2023) emphasized the latent value embedded in archival reports and argued for methods to convert scanned records into “living” data that can be searched, analyzed, and even reused for AI applications. Similarly, the National Academies of Sciences, Engineering, and Medicine (NASEM) (2022) underscored both the promise and complexity of applying AI to human health risk assessment through hosting a workshop under the request of the Center for Public Health and Environmental Assessment (CPHEA) within EPA’s Office of Research and Development (ORD). A central conclusion of all efforts was the necessity of coupling AI-assisted automation with human oversight to preserve transparency, interpretability, and scientific integrity.

Consistent with these perspectives, the CLEAR-Extract pipeline was intentionally designed as an AI-assisted (not AI-autonomous) workflow. High-volume, repetitive, and structurally complex tasks—such as OCR, table detection, and chemical table reconstruction—were delegated to AI systems, while tasks requiring contextual judgment, domain expertise, and decision making—such as document relevance assessment, quality control, and validation of reconstructed output—were reserved for human experts. This design advanced traditional human-centered data curation by strategically embedding human review as quality-assurance checkpoints. This design philosophy echoed findings from Dobler et al. (2025), who stated that large generative AI models can substantially expedite data analysis and coding tasks but require expert supervision to prevent errors, misinterpretation, or overconfidence in outputs. In the context of environmental data curation, such oversight is particularly critical given the heterogeneity of legacy documents and the potential downstream use of extracted data in exposure assessment and public health research.

More broadly, this project illustrated the paradigm described by Natarajan et al. (2025), who argued that many so-called “human-in-the-loop” systems were more accurately described as “AI-in-the-loop” (AI2L), where the human remains the central evaluator and decision-maker and the AI serves in a supportive, assistive manner. CLEAR-Extract embraced this AI2L philosophy, showing that the value depends not solely on AI algorithm accuracy but also on the quality of human-AI collaboration. By formalizing human governance as a core design principle, this study demonstrated a scalable and reproducible approach to integrating AI into environmental data curation. The resulting pipeline supported parallel review by multiple researchers, accommodated heterogeneous document formats, and could be adapted to other domains where large volumes of legacy documents need to be analyzed and transformed into reliable, reusable data resources.

### 5.4. Implication for FAIR data and environmental health research

This study advanced the FAIR (Findable, Accessible, Interoperable, Reusable) principles (Wilkinson et al. 2016) in database stewardship by converting previously inaccessible, static environmental documents into structured, machine-readable datasets. The transformation of heterogeneous VOCs tables into standardized, metadata-enriched formats improves the findability of chemical contaminants across brownfield sites, geospatial regions, and even laboratories. Housing the curated tables within a centralized, version-controlled data repository ensures accessibility for future environmental health research, decision-making, and targeted community-engaged investigation. By standardizing naming convention, field structure, and units, the data schema promotes interoperability by enabling integration with existing geospatial layers, exposure models, and demographic database. Finally, releasing the extraction logic, documentation, and standardized templates through this paper supports reusability by allowing other researchers and agencies to adapt the pipeline to their specific areas of interest.

### 5.5. Limitation

Several limitations and boundary conditions should be considered when interpreting the findings of this study. First, the feasibility of AI-assisted extraction and reconstruction was inherently constrained by the quality of the source documents. A small number of pages could not be reliably processed due to severe document quality limitations, reflecting intrinsic properties of legacy documentation rather than deficiencies in the pipeline design.

Second, the CLEAR-Extract pipeline was developed and evaluated using historical environmental assessment documents for brownfield sites, which were characterized by substantial heterogeneity in reporting practice, document formats, table structures, and variable naming conventions. While the pipeline was intentionally designed to accommodate such variability, its performance and generalizability may differ when applied to document corpora with dramatically different features, such as documents from another environmental domain. Adaptation to new contexts, such as different contaminants or expanded document corpora, may require modification of processing rules, keyword filters, and harmonization logic.

Third, this study focused on feasibility, scalability, and governance rather than exhaustive benchmarking of extraction accuracy against a gold standard. Quantitative performance metrics, such as AUC/ROC, precision, recall, etc., were intentionally secondary to demonstrating whether heterogeneous, legacy documents could be systematically transformed into a harmonized database under real-world constraints. Future work may extend this framework by incorporating formal accuracy benchmarks, uncertainty quantification, or comparative evaluations across alternative AI configurations.

Finally, this study was conducted on a limited set of addresses and scaling the pipeline to larger datasets could introduce additional challenges. For example, the use of AI-assisted components introduced computational cost considerations, which may influence scalability depending on available resources and system configuration. Also, the inclusion of human-in-the-loop evaluation, while essential for ensuring data quality, may require balancing between efficiency and scalability in large-scale applications.

## 6. Conclusion

In summary, this study developed CLEAR-Extract, a scalable AI-assisted human-in-the-loop pipeline that extracts, reconstructs, harmonizes, and standardizes tabular data from legacy environmental documents into a structured, analyzable database. By transforming unstructured and fragmented report data into a structured, analyzable database, this pipeline provided a practical pathway to convert static information into actionable evidence for future health risk assessments and sustainable redevelopment strategies. This work contributed directly to investigating and mitigating the ongoing public and environmental health crisis associated with brownfield sites. By producing a harmonized and standardized database, the pipeline enabled researchers at CLEAR to link the exposure to brownfield contamination with health risk assessments and epidemiological investigations, ensuring that the brownfield redevelopment program was well informed by the robust scientific evidence from the public health domain.

In this study, the pipeline achieved a 98% success rate in correctly processing addresses, including both successful reconstruction and correct identification of non-qualifying documents. The results underscore the pipeline’s ability to accommodate real-world variability in legacy environmental information while maintaining data integrity.

Beyond technical implementation, this study highlighted the irreplaceable role of human domain expertise in directing AI operations. This study demonstrated that human involvement remains essential in data processing work from initial document identification to intermediate quality evaluations and to final database harmonization and cleaning. The human judgment was the determining factor in many areas, such as establishing robust criteria, resolving ambiguities, and ensuring the feasibility and reliability, which were fundamental to the success of data processing. By explicitly positioning the human as the evaluator at each stage of the pipeline, this study demonstrated that human oversight is indispensable for trustworthy, contextually meaningful outcomes in AI-assisted environmental data processing.

Finally, by producing a harmonized, standardized VOC database aligned with FAIR principles, this work enhances the long-term findability, accessibility, interoperability, and reusability of environmental chemical data. The CLEAR-Extract pipeline provided a reproducible and governance-aware model for leveraging AI to modernize environmental data while preserving scientific rigor and accountability.

## Supplementary Material

Supp 1

Supplemental data for this article can be accessed online at https://doi.org/10.1080/2833373X.2026.2703318.

## Figures and Tables

**Table 1.** Harmonized VOC chemical data schema generated by the CLEAR-Extract.

#	Variable	Type	Len	Format	Description
1	File_FMT	Char	12	________	PDF file type
2	File_ID	Char	12	________	Unique identifier assigned to each PDF
3	PAGE_NUMBER	Num	8	________	Page number where chemical table was extracted
4	PARAMETE	Char	30	________	Chemical names
5	Unit_C	Char	20	________	Measurement unit
6	result_C	Char	20	________	Observed chemical results
7	RL_num	Num	8	________	Chemical detection limit on lower boundary
8	DILUTION_num	Num	8	________	Dilution factor applied during lab test
9	DATE_ANALYZED	Num	8	MMDDYY10.	Date the sample was analyzed
10	DATE_PREP	Num	8	MMDDYY10.	Date the sample was prepared

**Table 2.** Summary statistics of VOCs.

	Observed value (μg/kg)			Imputed value(μg/kg)^a^			Detectable indicator	
			
VOC chemical	*N*	Mean	Median	*N*	Mean	Median	*N*	%
**benzene**	312	3,066,546	1000	1586	981,753.3	30	1968	18
**ethylbenzene**	395	967,275.5	2200	1621	546,546.4	30	2002	22
**toluene**	297	24,212,188	2000	1601	5,052,919	30.5	1999	18
**Total xylenes**	498	4,863,203	3000	2414	1,912,332	75	3040	20
**Total VOCs**	1502	7,291,432	2000	7222	2,097,633	36	9009	20

a Non-detectable concentrations were imputed using LOD/2, where LOD is lower detection limit (e.g., RL_num, from Table 1).

## Data Availability

The original environmental reports analyzed in this study are publicly available from the Michigan Department of Environment, Great Lakes, and Energy (EGLE) Remediation Information Data Exchange (RIDE) website. The analytical dataset generated during this study was derived through data extraction, cleaning, standardization, and harmonization of these publicly available reports. The derived dataset has not been deposited in a public repository but may be available from the corresponding author upon reasonable request, subject to institutional policies, applicable data-sharing agreements, and funding requirements.
