# Supplementary material for: AI-assisted, human-in-the-loop pipeline for converting historical documents into structured database: a feasibility study of environmental application in Michigan
Source: Evid Based Toxicol. Author manuscript; Available in PMC 2026 Sep 18. (PMC13585084; doi:10.1080/2833373x.2026.2703318)
Supplement: Supplementary file 1 [file NIHMS2207181-supplement-Supp_1.pdf]

S1. STARD checklist for reporting of studies of diagnostic accuracy

**Section and Topic**	**Item #**		**On page #**
TITLE/ABSTRACT/ KEYWORDS	1	Identify the article as a study of diagnostic accuracy (recommend MeSH heading 'sensitivity and specificity').	1-2
INTRODUCTION	2	State the research questions or study aims, such as estimating diagnostic accuracy or comparing accuracy between tests or across participant groups.	3-5
METHODS			
Participants	3	The study population: The inclusion and exclusion criteria, setting and locations where data were collected.	5
	4	Participant recruitment: Was recruitment based on presenting symptoms, results from previous tests, or the fact that the participants had received the index tests or the reference standard?	DA
	5	Participant sampling: Was the study population a consecutive series of participants defined by the selection criteria in item 3 and 4? If not, specify how participants were further selected.	5
	6	Data collection: Was data collection planned before the index test and reference standard were performed (prospective study) or after (retrospective study)?	5
Test methods	7	The reference standard and its rationale.	6-8
	8	Technical specifications of material and methods involved including how and when measurements were taken, and/or cite references for index tests and reference standard.	6-8
	9	Definition of and rationale for the units, cut-offs and/or categories of the results of the index tests and the reference standard.	8-11
	10	The number, training and expertise of the persons executing and reading the index tests and the reference standard.	8-11
	11	Whether or not the readers of the index tests and reference standard were blind (masked) to the results of the other test and describe any other clinical information available to the readers.	DA
Statistical methods	12	Methods for calculating or comparing measures of diagnostic accuracy, and the statistical methods used to quantify uncertainty (e.g. 95% confidence intervals).	11-13
	13	Methods for calculating test reproducibility, if done.	DA
RESULTS			
Participants	14	When study was performed, including beginning and end dates of recruitment.	13
	15	Clinical and demographic characteristics of the study population (at least information on age, gender, spectrum of presenting symptoms).	DA
	16	The number of participants satisfying the criteria for inclusion who did or did not undergo the index tests and/or the reference standard; describe why participants failed to undergo either test (a flow diagram is strongly recommended).	14-16
Test results	17	Time-interval between the index tests and the reference standard, and any treatment administered in between.	DA
	18	Distribution of severity of disease (define criteria) in those with the target condition; other diagnoses in participants without the target condition.	DA
	19	A cross tabulation of the results of the index tests (including indeterminate and missing results) by the results of the reference standard; for continuous results, the distribution of the test results by the results of the reference standard.	16
	20	Any adverse events from performing the index tests or the reference standard.	DA
Estimates	21	Estimates of diagnostic accuracy and measures of statistical uncertainty (e.g. 95% confidence intervals).	15-16

	22	How indeterminate results, missing data and outliers of the index tests were handled.	5-8
	23	Estimates of variability of diagnostic accuracy between subgroups of participants, readers or centers, if done.	DA
	24	Estimates of test reproducibility, if done.	DA
DISCUSSION	25	Discuss the clinical applicability of the study findings.	16-18

*DA: Does Not Apply

S2. Supplementary Table 1: List of addresses, PDF and VOC status.

Commented [QZ1]: This will be placed in Supplementary Section.

Seq	PDF_Address	PDF	Pages	Rows	VOC IND	Min. Date Analyzed	Max. Date Analyzed
1	10905 Gratiot Ave	2	29	286	1	01/17/1994	01/26/1994
2	11187 Gratiot Ave	3	35	872	1	05/20/2006	05/29/2014
3	11545 Van Dyke St	8	72	4530	1	12/10/2020	08/15/2022
4	1160 S OAKWOOD	1	11	249	1	03/15/2010	03/18/2010
5	11621 Van Dyke St	1	16	517	1	03/17/2015	03/20/2015
6	11643 Hamilton Ave	1	27	1290	1	03/24/2015	03/28/2015
7	120 5th St	1	1	42	1	10/27/2021	10/27/2021
8	123 TEMPLE	1	26	508	1	01/28/2009	01/29/2009
9	1241 Fort St	1	18	604	1	05/07/2021	07/06/2021
10	13141 ROSA PARKS BLVD	7	93	3341	1	02/28/2001	11/04/2019
11	1320 MOUNT ELLIOTT ST	1	41	1173	1	10/02/2018	10/10/2018
12	14015 HAMILTON AVE	1	26	680	1	05/09/2014	05/13/2014
13	14243 Gratiot Ave	1	40	555	1	06/02/2005	06/11/2008
14	1500 E FERRY ST	1	24	709	1	11/13/2002	12/29/2002
15	15787 HARPER AVE	1	9	95	1	10/23/2009	10/23/2009
16	1600 E Warren Ave	6	310	16937	1	09/24/2001	06/18/2013
17	1601 ROBERT BRADBY DR	1	21	572	1	07/27/2012	08/23/2012
18	1601 Theodore St	1	21	366	1	03/31/2004	03/31/2008
19	16099 HAMILTON AVE	4	38	1576	1	02/23/2000	06/23/2014
20	17040 E WARREN AVE	2	7	315	1	02/16/1995	06/16/1997
21	17100 Van Dyke St	1	71	1012	1	08/31/1999	11/10/1999
22	17676 MOUNT ELLIOTT ST	2	36	1010	1	02/06/2018	02/28/2018
23	17699 Mount Elliott Street	3	124	4239	1	01/24/2022	12/16/2022
24	17900 WOODWARD AVE	2	38	1246	1	08/11/2003	03/29/2010
25	1881-1891 Gratiot Ave	2	46	1746	1	12/09/2013	01/23/2020
26	1891 E GRAND BLVD E	1	27	836	1	01/17/2020	01/23/2020
27	1891 East Grand Blvd	1	38	1291	1	01/17/2020	01/23/2020

**28**	19100 GLENDALE	1	138	4030	1	03/28/2006	03/28/2006
**29**	19100 JOHN R ST	2	4	77	0	05/28/2008	06/29/2008
**30**	1931 E Grand Blvd	1	16	577	1	08/01/2013	08/13/2013
**31**	1940 E Forest Ave	1	272	8868	1	07/02/2007	07/31/2008
**32**	1950 Medbury	2	14	170	0	01/08/1999	09/07/1999
**33**	2016 E Jefferson Ave	1	10	411	1	10/13/2021	10/18/2021
**34**	2102 SCOTTEN ST	3	28	1473	1	12/16/2013	08/12/2014
**35**	2150 E VERNOR HWY	1	12	290	1	06/11/2008	06/13/2008
**36**	216 S FORT ST	1	6	180	1	09/11/2012	09/11/2012
**37**	24637 Gratiot Ave	4	114	2672	1	04/04/2000	07/23/2013
**38**	2545 W GRAND BLVD	1	1	10	1	10/16/2009	10/16/2009
**39**	2600 Mack Ave	1	8	429	1	12/09/2013	12/10/2013
**40**	285 PIQUETTE ST	2	416	9458	1	09/29/2008	12/05/2008
**41**	2880 Scotten Street	1	12	137	1	05/13/1999	07/29/1999
**42**	301 Hosmer St	1	19	325	1	08/23/2001	08/23/2001
**43**	3163 CASS AVE	1	14	386	1	08/11/2003	08/14/2003
**44**	3168 Third Street	1	15	232	1	01/13/2015	01/16/2015
**45**	3333 Gratiot	1	3	110	1	10/11/2001	10/19/2001
**46**	3420 CASS AVE	1	3	96	1	04/24/2017	04/29/2017
**47**	3425 Gratiot	1	16	482	1	06/05/2005	06/14/2005
**48**	3652 Gratiot Ave	1	6	150	1	02/16/2021	02/19/2021
**49**	3700 HUMBOLDT ST	1	19	490	1	02/22/2007	03/08/2007
**50**	3711 E Vernor Hwy	2	18	791	1	08/01/2013	08/02/2013
**51**	3764 Charlevoix Rd	1	23	834	0	02/24/2016	02/26/2016
**52**	3800 Woodward Ave	1	9	174	1	01/22/2015	01/26/2015
**53**	3901 Grand River Ave	2	36	983	1	08/13/1982	01/28/2020
**54**	3901 Mount Elliott St	8	38	2546	1	08/05/2013	04/26/2016
**55**	3909 HASTINGS	1	53	747	1	07/20/2010	10/13/2010
**56**	398 St John St	2	21	650	1	11/16/1998	11/20/2014
**57**	4122 SAINT AUBIN ST	1	56	1363	1	11/21/2018	11/26/2018
**58**	4225 E DEQUINDRE	1	60	1072	1	08/02/2017	10/02/2017
**59**	4235 W Fort St	2	3	28	1	04/27/1994	05/09/1994
**60**	4819 FAIRVIEW ST	1	33	1152	1	06/02/2015	06/05/2015
**61**	5100 BRUSH ST	1	2	16	1	08/04/2008	08/04/2008
**62**	5133 TIREMAN ST	1	10	119	1	02/04/2013	04/05/2013
**63**	5427-5429 CHENE	1	3	85	1	10/10/2001	10/18/2001
**64**	5571 CHENE	1	2	76	0	10/11/2001	10/18/2001
**65**	5635 W Fort St	1	23	713	1	07/31/2012	08/08/2012
**66**	5761 Russell	1	77	2489	1	01/11/2018	01/16/2018

**67**	5778 Rivard	4	147	6275	1	06/02/2006	06/11/2020
**68**	5781 Russell	3	115	6342	1	06/07/2006	01/26/2018
**69**	5800 CASS AVE	1	15	230	1	10/01/2009	10/05/2009
**70**	5863 CHENE	1	4	150	1	10/11/2001	10/18/2001
**71**	5900 W WARREN AVE	1	34	1109	1	08/22/2008	08/29/2008
**72**	5901 Grand River Ave	1	23	607	1	09/25/2008	10/03/2008
**73**	5940 AVERY ST	1	18	455	1	05/14/2014	05/15/2014
**74**	5945 Avery	2	92	2231	1	05/08/2012	07/24/2014
**75**	5953 Avery	1	17	377	1	04/22/2014	05/08/2014
**76**	6051 HASTINGS ST	2	9	45	0	07/19/2005	09/09/2010
**77**	6110 CASS AVE	2	56	1310	1	09/10/2012	05/14/2023
**78**	6300 E VERNOR HWY	3	64	1804	1	10/27/1994	08/03/2013
**79**	6480 E 8 MILE RD	2	16	419	1	05/27/2008	08/25/2008
**80**	6480 East Jefferson Avenue	1	24	1107	1	06/14/2013	08/21/2013
**81**	6520 Hastings Street	2	106	2395	1	06/04/2001	12/19/2005
**82**	6600 Mount Elliott St	4	192	5129	1	11/06/1997	05/17/2012
**83**	6660 Mt Elliott St	1	229	3829	1	02/05/1999	12/12/1999
**84**	6841 E JEFFERSON AVE	2	28	488	1	08/15/1994	08/02/2013
**85**	7053 Gratiot Ave	1	21	109	1	07/01/1992	02/12/1993
**86**	7930 AGNES ST	1	52	1196	1	12/12/2012	10/29/2013
**87**	8003 Kercheval	4	137	4615	1	01/04/2016	02/07/2020
**88**	8121 GRATIOT AVE	1	5	122	1	01/24/2007	01/29/2007
**89**	9045 Vincent St	3	8	146	1	12/16/1997	03/02/2016
		**159**	**4270**	**131978**	**84**		
